# Comparison of Three DNA Isolation Methods and Two Sequencing Techniques for the Study of the Human Microbiota

**DOI:** 10.3390/life15040599

**Published:** 2025-04-04

**Authors:** Julio Plaza-Díaz, Mariana F. Fernández, Federico García, Natalia Chueca, Luis Fontana, Ana I. Álvarez-Mercado

**Affiliations:** 1 Institute of Biosanitary Research (ibs.GRANADA), San Cecilio University Clinical Hospital, 18012 Granada, Spain; jrplaza@ugr.es (J.P.-D.); marieta@ugr.es (M.F.F.); fegarcia@ugr.es (F.G.); nchueca@ugr.es (N.C.); 2 School of Health Sciences, International University of La Rioja, 26001 Logroño, Spain; 3 Spanish Consortium for Research on Epidemiology and Public Health (CIBERESP), 28029 Madrid, Spain; 4 Department of Radiology and Physical Medicine, School of Medicine, University of Granada, 18016 Granada, Spain; 5 Microbiology Unit, San Cecilio University Clinical Hospital, 18016 Granada, Spain; 6 Spanish Consortium for Research on Infectious Diseases (CIBERINFEC), 28029 Madrid, Spain; 7 Department of Biochemistry and Molecular Biology II, School of Pharmacy, University of Granada, 18071 Granada, Spain; 8 Institute of Nutrition and Food Technology “José Matáix”, Centre of Biomedical Research, University of Granada, 18016 Granada, Spain; 9 Department Pharmacology, School of Pharmacy, 18071 Granada, Spain

**Keywords:** breast cancer, breast microbiota, gut microbiota, extraction methods, microbial identification, shotgun, 16S rRNA gene amplification

## Abstract

Breast cancer is the most commonly diagnosed cancer in women and the second leading cause of female death. Altered interactions between the host and the gut microbiota appear to play an influential role in carcinogenesis. Several studies have shown different signatures of the gut microbiota in patients with breast cancer compared to healthy women. Currently, there is disagreement regarding the different DNA isolation and sequencing methodologies for studies on the human microbiota, given that they can influence the interpretation of the results obtained. The goal of this work was to compare (1) three different DNA extraction strategies to minimize the impact of human DNA, and (2) two sequencing strategies (16S rRNA and shotgun) to identify discrepancies in microbiome results. We made use of breast tissue and fecal samples from both healthy women and breast cancer patients who participated in the MICROMA study (reference NCT03885648). DNA was isolated by means of mechanical lysis, trypsin, or saponin. The amount of eukaryotic DNA isolated using the trypsin and saponin methods was lower compared to the mechanical lysis method (mechanical lysis, 89.11 ± 2.32%; trypsin method, 82.63 ± 1.23%; saponin method, 80.53 ± 4.09%). In samples with a predominance of prokaryotic cells, such as feces, 16S rRNA sequencing was the most advantageous approach. For other tissues, which are expected to have a more complex microbial composition, the need for an in-depth evaluation of the multifactorial interaction between the various components of the microbiota makes shotgun sequencing the most appropriate method. As for the three extraction methods evaluated, when sequencing samples other than stool, the trypsin method is the most convenient. For fecal samples, where contamination by host DNA is low, no prior treatment is necessary.

## 1. Introduction

### 1.1. Role of the Microbiome in Breast Cancer and the Importance of Microbiome Research

To maintain the homeostasis of the organism, humans need a stable and balanced microbiome or collection of microbes including bacteria, fungi, archaea, and viruses [1]. Alterations in the normal microbiota, referred to as dysbiosis, are related to a wide array of diseases including breast cancer [2]. As it occurs in the digestive tract, a specific microbiome is supposed to be beneficial to maintaining a healthy breast microenvironment. Evidence supports this hypothesis [3] since antibiotic treatment has resulted in breast tumor growth in mice [4,5] and humans [6,7]. Moreover, differences in gut microbiome profiles between benign and malignant tumors suggest that this profile is associated with the disease [2]. In a study reported by Ma et al. [8], 59 members of the microbiota differed in abundance between patients with breast cancer and those with benign breast disease. Patients with breast cancer exhibited lower relative abundances of *Firmicutes* (*Faecalibacterium*) and *Bacteroidetes* and increased abundances of *Verrucomicrobia*, *Proteobacteria*, *Actinobacteria*, *Bacillus*, *Enterobacter*, and *Staphylococcus*. In light of the fact that breast lesions can produce precursors of breast cancer or serve as risk markers for the disease, identifying microbial profiles associated with these lesions may contribute to early detection and prevent the development of cancer.

Interestingly, some studies suggest intestinal bacteria can reach the breast tissue through different pathways and promote tumorigenesis [9]. These observations are crucial given the fact that breast cancer is the most prevalent and the second cause of death for women [10]. However, the connection between the human microbiome and breast cancer has not yet been explored in detail [11].

### 1.2. Sequencing Strategies and Their Significance in Microbiome Studies

Metagenomic sequencing is an appropriate strategy for the study of the microbial community. The increasing use of metagenome next-generation sequencing (NGS) technologies presents both a challenge and an opportunity for expanding knowledge about the microbes that reside in the human body and their applicability to clinical practice [12]. Technological advances in NGS are resulting in a rapid increase in research aimed at analyzing complex microbiome environments. Such advances provide longer read lengths and paired-sequencing reads to identify microbial communities better [13]. To date, sequencing-based microbiome methods have greatly enriched our understanding of the microbe population of the human body in both health and disease and have contributed to the expansion of our view of microbes in polymicrobial communities and infections [14]. Currently, there are several NGS platforms: The Roche 454 Genome Sequencer FLX System, the Illumina HiSeq and MiqSeq Systems, the Ion Torrent, the Applied Biosystems SOLiD sequencer, and Oxford Nanopore Technologies. However, some issues still need to be solved: (1) Existing sequencing technologies display platform-specific biases depending on the run mode and chemistry. These biases affect the read length, data throughput, guanine cytosine (GC) coverage bias, error rates, and the ability to resolve repetitive genomic elements [15]. (2) Due to the large diversity of generated data, the possibility to produce realistic benchmark datasets for particular experimental setups is challenging and essential. (3) Analyses are often hampered by overwhelming quantities of human DNA, yielding only a small proportion of microbial reads for analysis [14]. (4) Finally, the cost of metagenomic analysis per sample with high human-to-microbial DNA ratios could be high when considering the depths required for the thorough investigation of the microbiome in a large number of samples [16].

Nowadays, two extensively used metagenome sequencing strategies are shotgun and the small subunit (SSU) rRNA genes, namely the 16S rRNA. Both are used to catalog the human microbiome in health and disease and to study microbial communities of medical, pharmaceutical, or biotechnological relevance [17]. 16S rRNA gene sequencing identifies and quantifies species or amplicon sequence variants (ASVs) [18]. This methodology has greatly expanded our understanding of microorganism diversity leading to the creation of some public databases like the National Center for Biotechnology info (NCBI) GenBank, SILVA [19], the Ribosomal Information Project (RDP) [20], and Greengenes [21]. Because the 16S rRNA gene is universal in bacteria, this method presents some benefits: (1) the sequencing of a short (~1500 base pairs) region is relatively economical and fast, and (2) the common use of the same target gene across multiple studies increases accuracy in meta-analysis.

By contrast, although the V3 and V4 hypervariable regions of the bacterial 16S rRNA can be used to distinguish taxa, this method provides uneven resolution of the taxonomic spectrum. In addition, variation in the copy number of this gene influences abundance estimates. Another disadvantage of this method is that the accuracy of PCR approaches is reduced because of primer bias and chimeras. Moreover, PCR-based databases and primer analysis tools overestimate primer coverage [22].

Shotgun is a method used for analyzing random DNA fragments ranging from 100 to 1000 base pairs. DNA is fragmented into numerous small segments and sequenced to obtain reads. Multiple overlapping reads for the target DNA are obtained by performing several rounds of this fragmentation and sequencing separately. Finally, the sequences are assembled to give an overall sequence [23]. Shotgun sequencing was one of the precursor technologies that led to whole genome sequencing. Metagenomic shotgun sequencing appears to be a powerful tool for identifying causative disease agents in human patients. This technique also allows the detection of different types of microbes other than bacteria. In addition, the limit of detection of this method is modulated by sequencing depth, read length, and data accuracy. This allows microbes to be detected to obtain more complete functional representations. However, one of its main limitations is its cost and the fact that the datasets obtained from microbial communities are very diverse, not only due to the natural variation of biological systems but also due to differences in laboratory protocols, the number of replications, and sequencing technologies [24].

### 1.3. Challenges in Microbiome Research and Rationale

Some challenges still need to be overcome: (1) Shotgun metagenomic datasets from microbial communities are highly variable, not only due to the natural variation of the underlying biological systems but also due to differences in laboratory protocols, replicate numbers, and sequencing technologies; (2) overwhelming amounts of human DNA lead to potential confounding and a small proportion of microbial reads for analysis.

Several studies have shown that DNA extraction methods play a crucial role in bacterial community detection [25,26,27,28,29]. There are several steps involved in DNA extraction, including weighing, homogenization, lysis of bacteria, and purification of DNA, each of which still requires improvement and guidelines. The standard weighing procedure, for example, may be time consuming and tedious for collecting the same volume of fecal material for all samples. There is also a possibility that homogenization of the sample could impact the amount of detectable bacteria [30,31].

This work is part of an ongoing clinical case–control study (Identifier NCT03885648) in which the mammary and fecal microbiota from healthy women and breast cancer patients are being compared. Although it is nowadays known that breast tissue is not sterile, we expected the amount of microbial DNA to be low. In addition, the amount of breast tissue available was limited. Therefore, we decided that the extraction method should be optimized by comparing several protocols. Some current solutions to eliminate host contamination include the use of microbeads, but these methods require a larger sample size and are more expensive. On the other hand, although the comparison of extraction methods has been reported in recent years, the available comparison between protocols other than stool are very limited.

In the present study, we aimed to compare (1) three different DNA extraction strategies to minimize the impact of human DNA, and (2) two sequencing strategies (16S rRNA and shotgun) to identify discrepancies in microbiome results. For this purpose, we took advantage of fecal and mammary tissue samples obtained in an ongoing case–control clinical study from both healthy and breast cancer-affected women (MICROMA study) [2,32].

## 2. Materials and Methods

### 2.1. Design

This work is part of an ongoing case–control study registered at ClinicalTrials.gov (accessed on 30 September 2017) (Identifier NCT03885648) and described elsewhere [2,32]. Briefly, cases were women with stages I and II breast cancer. Controls were women who had breast augmentation or reduction surgery.

### 2.2. Biological Samples

Breast tissue and stool samples from cases and controls were collected as detailed in [2]. Samples were obtained from the Unit of Mammary Pathology, General Surgery Service at San Cecilio University Hospital (Granada, Spain). Breast tissue samples were obtained at the time of surgery from a marginal zone and distant from the tumor (3–5 cm), in the case of women with breast cancer, and any available breast tissue for women with breast reduction or enlargement. Stool samples were collected from healthy women and women with breast cancer in a time window of 1 to 3 days before the breast tissue collection [2].

### 2.3. DNA Isolation Methods

#### 2.3.1. Sample Lysis

For breast tissue, frozen samples were first ground in a mortar. Twenty-five milligrams of sample (feces and breast tissue) were homogenized in PBS by vortex shaking at maximum speed. Later on, additional mechanical lysis with pathogen lysis tubes L (recommended for fungi and some resistant bacteria) and S (recommended for bacteria) (Qiagen, Barcelona, Spain) was carried out. Three different methodologies (described below) were tested for DNA isolation.

Method A. This method involves mechanical lysis with pathogen tubes L and S as described. No additional treatment was performed.

Method B. Selective removal of human DNA from metagenomic DNA samples was carried out following the methodology described by Hunter et al. (2011) [33]. Briefly, after the mechanical lysis described above, samples were centrifuged at 13,000× *g* for 2 min. The supernatant was discarded and the pellet containing all cells was suspended in 1 mL TrypZean (Sigma T3449, St. Louis, MO, USA) containing 0.05% Tween-20, and subsequently incubated at 37 °C for 70 min with gentle shaking in a water bath. Samples were again briefly vortexed and centrifuged at 5000× *g* for 2 min. The supernatant was transferred into a new tube and centrifuged at 13,000× *g* for 10 min. The pellet was then suspended in 0.4 mL PBS. Then, the described additional mechanical lysis was performed using pathogen lysis tubes L and S.

**Figure 1:**
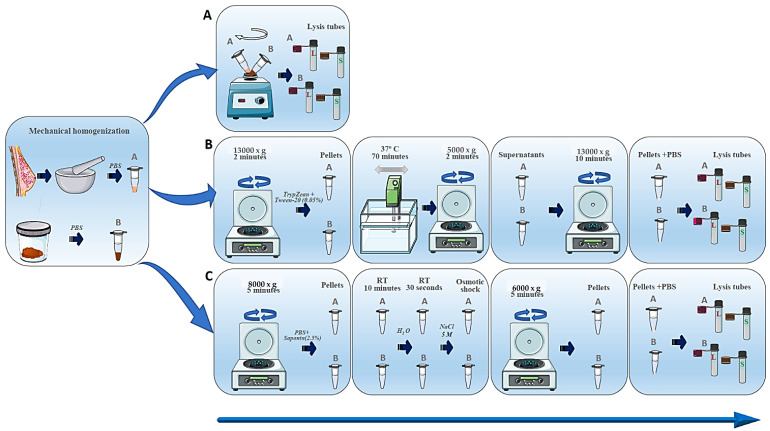
Schematic representation of different extraction methods used in this work. (**A**) Mechanical lysis; (**B**) trypsin method; (**C**) saponin method. PBS: phosphate-buffered saline; RT: room temperature.

Method C. This is a modified saponin-based differential lysis method. Samples were centrifuged at 8000× *g* for 5 min, then the supernatant was carefully removed and the pellet suspended in 250 μL of PBS. Saponin was applied to a final concentration of 2.5%, mixed well, and incubated at room temperature for 10 min to promote host cell lysis. Following this incubation, 350 μL of water was added and incubation was continued at room temperature for 30 s. After this, 12 μL of 5 M NaCl was added to deliver an osmotic shock, lysing the damaged host cells. Samples were next centrifuged at 6000× *g* for 5 min; the supernatant was removed and the pellet re-suspended in 100 μL of PBS. Further mechanical lysis was performed with pathogen lysis tubes L and S. Figure 1 summarizes the aforementioned methods.

#### 2.3.2. DNA Extraction

The QIAamp^®^ cador^®^ Pathogen Mini kit (Qiagen, Barcelona, Spain) was used to isolate genomic DNA according to the manufacturer’s instructions. The concentration and purity of DNA were determined using a NanoDrop ND-1000 (Thermo Fisher Scientific, Waltham, MA, USA).

### 2.4. DNA Sequencing

Two different metagenome sequencing strategies were carried out in this study: the 16S rRNA method and shotgun sequencing.

16S rRNA method. The V3 and V4 hypervariable regions of the bacterial 16S rRNA gene were PCR amplified using the following primers: 16S Amplicon PCR Forward Primer (5′-TCGTCGGCAGCGTCAGATGTGTATAAGAGACAGCCTACGGGNGGCWGAG-3′) and 16S Amplicon PCR Reverse Primer (5′-GTCTCGTGGGCTCGGAGATGTGTATAAGAGACAGGACTACHVGGGTATCTAATCC-3′) [34]. A 25 μL reaction volume was used to perform all PCR reactions containing 12.5 μL 2X KAPA HiFi Hotstart ready mix (KAPA Biosystems, Woburn, MA, USA), 5 μL of each forward and reverse primers (1 μM), and 2.5 μL of extracted DNA (10 ng) under the following cycling conditions: initial denaturation at 95 °C for 3 min, followed by cycles of denaturation at 95 °C for 30 s, annealing at 55 °C for 30 s, and elongation at 72 °C for 30 s, followed by a final extension at 72 °C for 5 min. The 16S V3 and V4 amplicons were purified with AMPure XP beads (Beckman Coulter, Indianapolis, IN, USA) from free primers and primer dimer species. Next, the index PCR was conducted, in which dual indices and Illumina sequencing adapters were attached with the Nextera XT Index Kit (Illumina, San Diego, CA, USA), PCR conditions were: 95 °C for 3 min; 8 cycles of 95 °C for 30 s, 55 °C, 72 °C for 30 s; 72 °C for 5 min, and hold at 4 °C. Before quantification, the pooled PCR products were purified with AMPure XP beads (Beckman Coulter, Indianapolis, IN, USA). Using a paired-end (2 × 300 nt) Illumina MiSeq sequencing system (Illumina, San Diego, CA, USA), the amplicons were sequenced at MiSeq (Illumina, San Diego, CA, USA) [2,32].

Shotgun method. Metagenomic libraries were constructed with Nextera XT DNA Library Preparation Kit (Illumina, San Diego, CA, USA). For library construction, we used 100 pg input DNA and the amplicon tagment mix (ATM) in Nextera XT was diluted 1:10 in nuclease-free water. The tagmentation reaction mixture consisted of TD buffer (10 μL), input DNA (5 μL), and diluted ATM (5 µL). Following the manufacturer’s protocol, PCR cycles were conducted for 100 pg DNA for 17 cycles. The amplified libraries were purified with AMPure XP (Beckman Coulter, Indianapolis, IN, USA). The quality of the purified libraries was assessed using an Agilent High Sensitivity DNA Kit on an Agilent 2100 Bioanalyzer (Santa Clara, CA, USA). The sequencing libraries were further quantified using the KAPA Library Quantification Kit. The metagenomics libraries were mixed with PhiX Control v3 (Illumina, San Diego, CA, USA) at a ratio of 9:1 and sequenced with an Illumina MiSeq Reagent Kit v3 (600 cycles) (Illumina, San Diego, CA, USA) [2,32].

### 2.5. Bioinformatic Analyses

#### 2.5.1. 16S rRNA Method

Raw sequences were demultiplexed with Illumina’s bcl2fastq2 Conversion Software v2.20 and raw data were imported into open-source software QIIME 2 2020.8 [35] with the q2-tools-import script with PairedEndFastqManifestPhred33 input format. Using the V3-V4 16S rRNA sequencing method, reads were generated. To obtain an abundance distribution of sequence variances that have a difference of one nucleotide, we applied DADA2 [18], which provides a quality-aware model of Illumina amplicon errors. Using the q2-dada2-denoise script, forward reads were truncated at position 288 and trimmed at position 6 after retrieving quality scores. The reverse reads were truncated at position 220 and trimmed at position 7. As part of the q2-dada2-denoise algorithm, chimeras were removed with the “consensus” filter, in which chimeras were detected in samples individually and sequences found chimeric in a sufficient fraction of samples were eliminated. As part of this process, forward and reverse reads were also merged. Using MAFFT [36] via q2-alignment, all amplicon sequence variants (ASVs) were aligned and a phylogeny was constructed with FASTTREE2 (via q2-phylogeny) [37]. Based on the SILVA 16S V3-V4 v132_99 [17], ASVs were assigned taxonomy using the Naive Bayes taxonomy classifier (via q2-feature-classifier) [38]. Samples with fewer than 10,000 reads were pruned during the data filtering process.

#### 2.5.2. Shotgun Method

Raw sequences were analyzed with MetaPhlAn 3.0 (Metagenomic Phylogenetic Analysis), a computational tool for profiling the composition of microbial communities from metagenomic shotgun sequencing data [39].

### 2.6. Statistic Analysis

The median and range were used to express the results. *p* < 0.05 was considered statistically significant. Variables that were not normally distributed were log-transformed, and outliers were removed (without achieving a loss of value from samples of up to 15%). To ensure a clear understanding of the data, the values were presented untransformed. To assess differences in the relative abundance of bacteria (phylum and genus), the U Mann–Whitney test was applied. Unless otherwise specified, data are presented as mean ± SEM. A Kruskal–Wallis test determined statistical significance. Significance was adjusted by the Bonferroni correction for multiple tests. For the alpha indices, two-tailed Student’s *t*-tests were performed to compare each of the indices determined with the samples sequenced by 16S rRNA or shotgun.

Rivera-Pinto analysis identifies microbial signatures, i.e., groups of microbes that can predict particular phenotypes. Diagnosing, prognosticating, or predicting therapeutic responses is possible using the microbial signatures associated with an individual’s microbiome. It is possible to identify microbial signatures by modeling the response variable and selecting taxa that yield the highest classification or prediction accuracy level. With the Rivera-Pinto method and the selbal algorithm, we evaluated specific signatures at the phylum and genus levels to select a sparse model that adequately explains the response variable [40].

## 3. Results

This study was carried out on stool and mammary tissue samples from patients with breast cancer (cases) and healthy women (controls) who participated in the MICROMA study [2,24].

### 3.1. Human DNA Depletion

**Figure 2:**
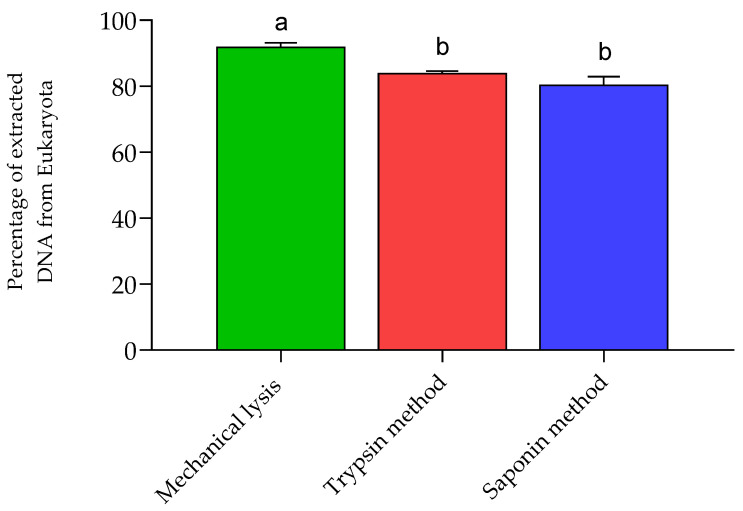
Percentage of DNA isolated from eukaryotic cells in breast tissue. Labeled medians with identical letters are not significant. Different letters mean significant differences (*p* < 0.05). Mechanical lysis vs. trypsin method, *p* = 0.0031; mechanical lysis vs. saponin method *p* = 0.0006; trypsin method vs. saponin method *p* = 0.2571.

To assess the efficiency of depleting human DNA from samples, we performed metagenomic sequencing of all DNA extracts, then calculated the proportion of eukaryotes to total sequence reads. Our results indicate a significant decrease in the amount of DNA from eukaryotic cells in the trypsin and saponin methods compared to the mechanical lysis method (Figure 2). We were able to detect archaea in our shotgun metagenomic sequencing.

### 3.2. 16S rRNA Sequencing

The abundance of the phylum *Actinomycetota* was greater in the fecal samples of healthy women treated with saponin compared to those treated with trypsin and mechanically. In comparison with the mechanical and saponin DNA isolation methods, the trypsin method resulted in a significant increase in the relative abundance of the phylum *Bacteroidota*. The phylum *Bacillota* was significantly more abundant when DNA was isolated by mechanical lysis or with saponin in comparison with the trypsin method. The *Pseudomonadota* and *Verrucomicrobiota* phyla were less abundant when the DNA was isolated using saponin compared to the mechanical lysis method. Furthermore, the DNA isolation method that resulted in the lowest yield in the phylum *Verrucomicrobiota* was trypsin (Table 1A). 

The abundance of phyla *Bacteroidota*, *Pseudomonadota*, and *Bacillota* in breast tissue samples from healthy women differed significantly. *Bacteroidota* and *Pseudomonadota* were significantly higher with the mechanical lysis and trypsin methods compared with the saponin method, whereas *Bacillota* was significantly higher with the saponin method than with the mechanical lysis (Table 1A).

The methods of DNA isolation using mechanical lysis and trypsin produced similar results, while the results obtained with the saponin method were very different to the previous two. Due to this and the fact that the latter method includes more intermediate steps and is therefore longer, it was decided to continue with the first two methods (Table 1B).

We found significant differences in the *Bacteroidota* and *Verrucomicrobiota* phyla in the feces of patients with breast cancer. In these two phyla, the trypsin method resulted in significantly higher relative abundances compared with the mechanical lysis method (Table 1B). In breast tissue samples, *Bacteroidota* was the only phylum that changed, with a greater abundance with the trypsin method compared with the mechanical lysis (Table 1B).

### 3.3. Shotgun

*Pseudomonadota*, *Actinomycetota*, and *Bacteroidota* fecal abundances were significantly higher in trypsin-treated samples than in mechanically analyzed samples from control patients. In the shotgun analysis of breast tissue samples from breast cancer patients and control women, no identifications were found for the mechanical lysis method (Table 2). On the contrary, identifications were made for both control and breast cancer women using the trypsin method (Table 2). Significant statistical changes were observed in the feces of breast cancer patients: the abundance of the *Actinomycetota* and *Pseudomonadota* phyla was significantly greater with trypsin-treated samples than with mechanically analyzed samples (Table 2).

### 3.4. Diversity Index Comparison

**Figure 3:**
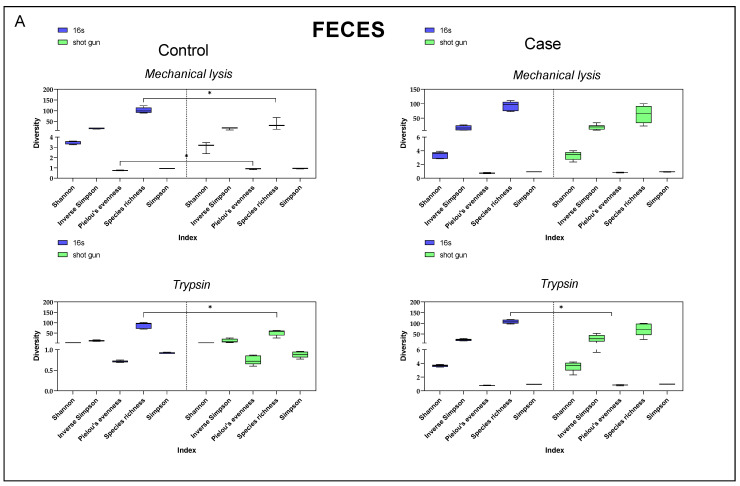

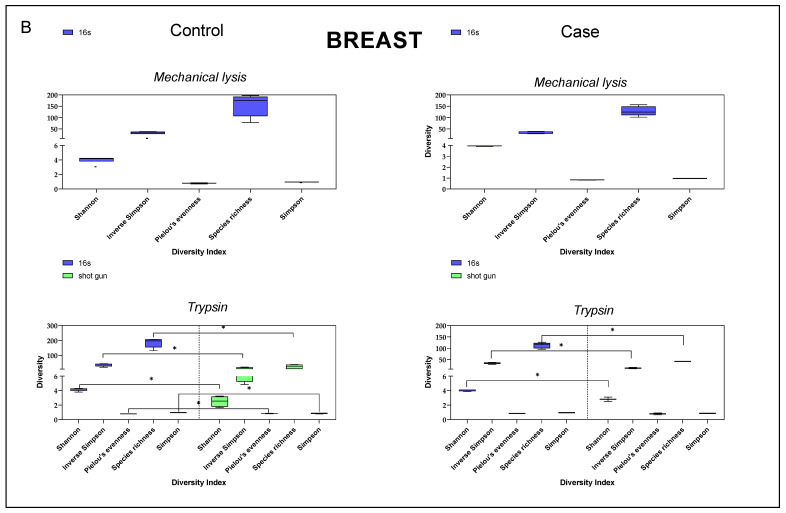
Alpha diversity indices for stool samples (**A**) and breast tissue samples (**B**). Figure shows various alpha diversity indices for samples whose DNA was isolated using mechanical lysis or trypsin methods, and sequenced by 16S rRNA or shotgun methods. * *p* < 0.05. Cases (n = 5), controls (n = 5).

When comparing different alpha diversity indices between samples sequenced by 16S rRNA or shotgun and extracted with mechanical lysis or trypsin, no significant differences were observed in the stool samples in four of the five indices determined (Figure 3A).

On the contrary, in the case of breast tissue samples, we were unable to determine any of these indices when the DNA was extracted by mechanical lysis and sequenced by shotgun. Significant differences in the diversity indices were observed in both cases and controls for both 16S rRNA and shotgun sequencing methods when DNA isolation was carried out with trypsin (Figure 3B).

### 3.5. Microbiome Balance 

**Figure 4:**
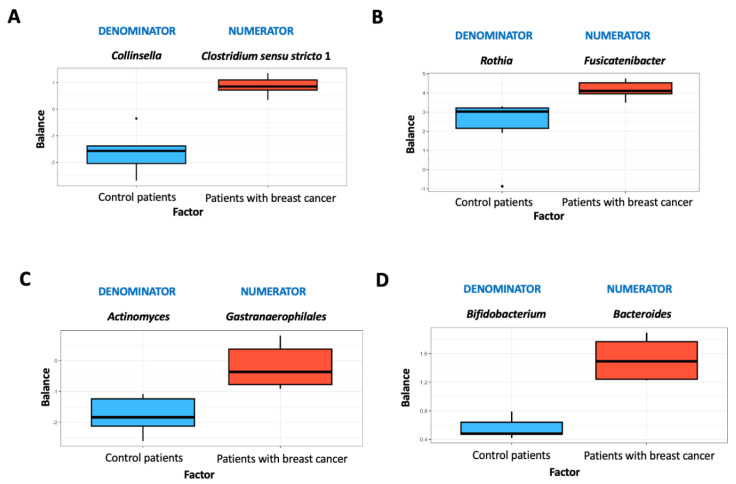
Microbiome balance for 16S rRNA method. Distribution of balance scores obtained with 16S rRNA analysis using mechanical lysis in feces samples (**A**) and breast samples (**B**), and with 16S rRNA analysis using trypsin method in feces samples (**C**) and breast samples (**D**). Patients with breast cancer (n = 5), controls (n = 5).

**Figure 5:**
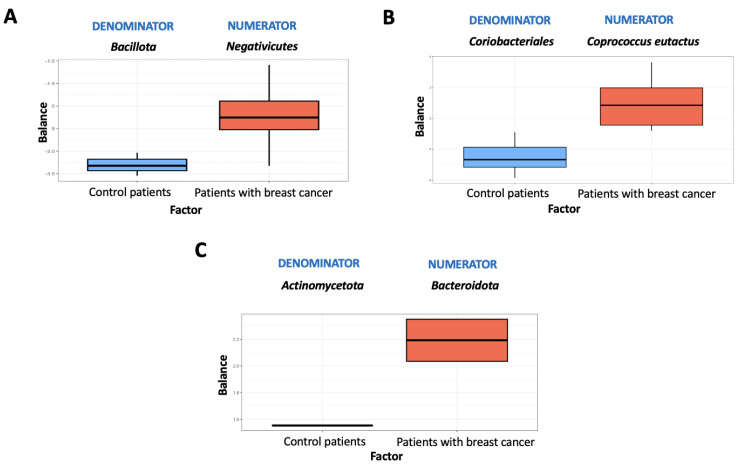
Microbiome balance for shotgun method. Distribution of balance scores obtained with shotgun analysis using mechanical lysis in feces samples (**A**) and with shotgun analysis using trypsin method in fecal samples (**B**) and breast samples (**C**). Patients with breast cancer (n = 5), controls (n = 5).

An overview of microbial balances for stool and mammary tissue samples is provided in Figure 4 and Figure 5. These figures depict the distribution of balance scores obtained with both sequencing techniques. As a result of the 16S rRNA analysis and using mechanical lysis in feces samples, *Collinsella* had lower balance scores than *Clostridium sensu stricto* 1 (Figure 4A).

On the contrary, *Clostridium sensu stricto* 1 was more prevalent in patients with breast cancer than in control women (Figure 4A). Using the mechanical lysis method and 16S rRNA sequencing, the breast samples were characterized by lower *Rothia* scores than *Fusicatenibacter* scores (Figure 4B). *Rothia* was the most commonly associated genus in healthy women (Figure 4B). In the case of the trypsin method in feces samples, *Gastranaerophilales* had higher scores than *Actinomyces*, the latter being more associated with control women (Figure 4C). The levels of *Bacteroides* were higher in breast samples treated with trypsin compared to *Bifidobacterium*. The presence of *Bacteroides* was associated with breast cancer patients (Figure 4D).

For mechanical lysis in feces samples, *Bacillota* and *Negativicutes* were the main taxa detected (Figure 5A). Using the trypsin method in feces samples, the order *Coriobacteriales* had lower scores than *Coprococcus eutactus*, the latter being more associated with patients with breast cancer (Figure 5B). Breast samples treated with trypsin contained higher levels of *Bacteroidota* than *Actinomycetota*. Patients with breast cancer were associated with the presence of the phylum *Bacteroidota* (Figure 5C).

## 4. Discussion

Technological advancements in NGS are rapidly expanding research into analyzing complex microbiome environments and revolutionizing microbiology [41]. Bioinformatics will inevitably be applied in this transition from a purely laboratory task to a computational one. Conversely, the possibility of conducting metagenomics studies quickly and at a reduced cost may facilitate faster disease diagnosis [42]. In order to establish a relationship between health and disease and develop an effective treatment for infectious diseases, it is essential to have a comprehensive understanding of epidemiology, virulence mechanisms, and host–organism interactions [43], with the human microbiota playing a key role in this scenario.

The present work makes use of breast tissue and fecal samples from both healthy women and breast cancer patients who participated in the MICROMA study (Identifier NCT03885648) [2,32]. We compared the effectiveness of various DNA extraction and genome sequencing strategies to identify possible discrepancies in the microbiome profile, as well as evaluated the precision and accuracy of microbial identification to improve diagnostic and treatment tools. Since human tissue analysis is often hampered by overwhelming quantities of human DNA [14], we also aimed to minimize the impact of human DNA in the samples. With this purpose, we compared the outcomes from three distinct extraction methods—namely, mechanical lysis, mechanical lysis accompanied by trypsin digestion, and a modified saponin-based differential lysis method. Saponin was selected since detergents, including saponin, are frequently used in cell lysis before DNA extraction [44,45]. Our results showed that the saponin method yielded the most divergent results. This finding, in conjunction with the observation that this method involves a greater number of intermediate steps and consequently exposes the process to a heightened risk of experimental inaccuracy, led to the decision to exclude it from subsequent experiments and continue the study without this method.

Concerning the trypsin method, it is known that this enzyme catalyzes the hydrolysis of peptide bonds, breaking down proteins into smaller peptides [46]. The use of trypsin to clear DNA is not new and has been previously reported, for instance, in forensic DNA analysis of bone samples before DNA isolation [47] and for preparing DNA from formalin-fixed paraffin-embedded samples for methylation analysis [48].

We are aware of the fact that alternative extraction methodologies may be employed for the optimization of DNA isolation by, for example, bead beating [49]. However, the implementation of this process produces a considerable increase in the expense of the sequencing process, whilst concomitantly necessitating a greater volume of samples, a requirement that is not universally met, particularly in the context of samples of human origin.

Although each person harbors a distinctive gut microbiota composition, the overall structure conforms to patterns that are repeated in different individuals (enterotypes). The enterotype concept suggests that the microbial ecosystem in the human gut forms internal states of symbiosis between the different members of the microbial community, probably determined by the very metabolic or social networks in which they are integrated. These interactions explain the stability and resilience of an ecosystem subject to fluctuations [50] and make neccesary consider all the components of the microbiota. Having this in mind, in stool samples we did not find a significant influence of tripsin in the overall picture of microbial diversity, where more than 90% of the microbial DNA is from bacteria. In contrast, our results for breast tissue samples were different since human DNA is predominant. By eliminating human DNA, one can better discern the microbial diversity althought this could result in the loss of valuable sequencing material.

Overall, in the present study, we confirmed that the extraction method used to identify microbial DNA sequences significantly influences the results. We also found that in human samples such as breast tissue, where microbial DNA is not predominant, the trypsin method was useful because it decreased the amount of human DNA and therefore facilitated sequencing.

On the other hand, the results of Rivera-Pinto’s analysis demonstrated different phyla- and genera-specific signatures. This finding is of great interest, since predicting particular phenotypes (based on the microbial signatures associated with an individual’s microbiome) is crucial in the diagnosis, prognosis, and therapeutic responses. Indeed, our results corroborate the fact that strategies to obtain and analyze metagenomic data still need a wide effort of standardization and validation to get comparable results and guarantee that the extensive amount of data being produced is reliable.

Given that the 16S rRNA method is specific to bacteria, it presents a reduced risk of host contamination [41]. Due to extensive reference databases and computational error correction tools, 16S rRNA sequencing also reduces the risk of false positives; however, the likelihood of false positives increases as sample biomass decreases [51]. In contrast, shotgun metagenomic sequencing represents an evolutionary precursor to whole genome sequencing. The technology is a highly effective means of identifying the causative agents of human diseases. However, a significant limitation of this method is the fact that the presence of a vast quantity of human DNA may result in the potential for confounding and a comparatively low proportion of microbial reads for analysis. This may explain the lack of data obtained for shotgun sequencing in breast tissue samples. With the 16S rRNA sequencing, however, the bacterial population is amplified and, consequently, human DNA is eliminated and microbial identification is facilitated. To the best of our knowledge, the majority of studies that use mammary tissue utilize 16S rRNA sequencing [52], and therefore there is no clear picture of the mammary microbiome.

In summary, this study confirms that DNA extraction and sequencing methods are critical in microbiota studies, as they can influence the interpretation of results. There are many aspects that remain to be resolved, so some recommendations that can be made for future research in this field would be as follows.

With regard to DNA extraction, it is necessary to standardize protocols to guarantee reproducibility. Variability in extraction methods can affect microbial composition, as our work suggests. Possible biases would also have to be evaluated (for example, comparing different commercial kits and homemade methods to determine which ones best preserve microbial diversity, avoiding the over- or under-representation of certain taxa) and protocols adapted to different types of samples to maximize the recovery of quality DNA. The elimination of contaminants is also a pending issue: it is necessary to implement negative controls and the use of DNA-free reagents to avoid contamination.

As for sequencing, the most common bioinformatic tools used to study microbial ecology include QIIME pipelines [53], Mothur software [54], and R packages, such as vegan [55]; phyloseq [56], DADA2 [18], and RAM [57]. Alpha/beta diversity metrics are calculated using the vegan R package [55]. It is of course important to make a comparison of the various existing platforms, but we think that these studies would benefit from multi-omic approaches to obtain a more complete picture of microbial function. Finally, although we are aware that it is a work in progress, there is a need to continue refining reference databases, bioinformatics tools and algorithms for genome reconstruction to minimize errors in species assignment.

The resolution of these pending issues is crucial for clinical research related to the human microbiota as they influence the accuracy of disease diagnosis, the personalization of and response to treatments, and the development of new therapies, among others.

## 5. Conclusions

In conclusion, in samples with a predominance of prokaryotic cells, such as feces, 16S rRNA sequencing is the most advantageous approach. For other tissues, which are expected to have a more complex microbial composition, the need for an in-depth evaluation of the multifactorial interaction between the various components of the microbiota makes shotgun sequencing the most appropriate sequencing method. As for the three extraction methods evaluated, when sequencing samples other than stool, the trypsin method is the most convenient. For fecal samples, where contamination by host DNA is low, no prior treatment is necessary.

## Figures and Tables

**Table 1:** (**A**) Identification of bacterial DNA based on 16S rRNA gene using three extraction methods in healthy women. (**B**) Identification of bacterial DNA based on 16S rRNA gene using three extraction methods in patients with breast cancer.

(**A**)						
**Phylum**	**Healthy Women (Controls)**					
	**Feces**			**Breast**		
	**A (n = 5)**	**B (n = 5)**	**C (n = 5)**	**A (n = 5)**	**B (n = 5)**	**C (n = 5)**
*Actinomycetota*	4.7 (0.3–13.8) ^a^	7.8 (2.7–10.7) ^a^	14.4 (13.5–17.5) ^b^	5.7 (4.8–6.2)	7.3 (5.6–9.9)	6.6 (5.0–8.0)
*Bacteroidota*	6.3 (3.0–22.8) ^a^	27.2 (15.2–33.9) ^b^	6.6 (2.6–22.7) ^a^	16.6 (9.4–19.0) ^a^	15.9 (6.2–19.2) ^a^	8.9 (7.1–11.3) ^b^
*Bacillota*	74.6 (69.7–87.0) ^a^	62.5 (48.7–72.9) ^b^	74.1 (63.1–79.6) ^ab^	57.5 (46.2–63.7) ^a^	64.6 (52.3–67.6) ^ab^	71.7 (60.0–78.6) ^b^
*Fusobacteria*	0 (0–0.03)	0 (0–0.2)	0 (0–0)	0 (0–0.04)	0 (0–0.1)	0 (0–0.01)
*Pseudomonadota*	2.0 (0.2–2.4) ^a^	0.6 (0.3–1.9) ^ab^	0.3 (0.1–1.3) ^b^	4.7 (3.6–10.0) ^a^	4.8 (3.7–12.6) ^a^	2.6 (0.5–2.8) ^b^
*Verrucomicrobiota*	2.3 (0–6.8) ^a^	0 (0–26.5) ^b^	0.02 (0–8.0) ^b^	2.3 (0.8–3.7)	3.0 (0.2–4.1)	1.5 (1.1–3.1)
Unassigned	0 (0–0)	0 (0–0)	0 (0–0)	0.04 (0–0.3)	0 (0–0.5)	0.09 (0–0.2)
(**B**)						
**Phylum**	**Patients with Breast Cancer (Cases)**					
	**Feces**				**Breast**	
	**A (n = 5)**		**B (n = 5)**		**A (n = 5)**	**B (n = 5)**
*Actinomycetota*	1.6 (1.1–29.0)		5.0 (3.5–7.2)		4.9 (1.9–6.0)	3.1 (1.5–5.1)
*Bacteroidota*	9.6 (0.8–32.0) ^a^		17.3 (3.7–24.6) ^b^		18.3 (16.1–18.9) ^a^	19.4 (18.4–23.5) ^b^
*Bacillota*	68.2 (52.8–95.0)		72.4 (58.0–87.7)		63.0 (57.7–70.2)	66.9 (60.0–69.2)
*Fusobacteria*	0 (0–0.01)		0 (0–0)		0 (0–0.1)	0.08 (0–0.3)
*Pseudomonadota*	2.0 (0.9–7.7)		1.2 (0.3–7.6)		4.6 (3.1–8.2)	4.2 (2.5–9.1)
*Verrucomicrobiota*	0.9 (0–6.3) ^a^		5.2 (0.06–14.2) ^b^		2.3 (1.5–3.0)	3.0 (1.9–3.5)
Unassigned	0 (0–0)		0 (0–0)		0.04 (0.02–0.2)	0.01 (0–0.02)

(**A**) A. Mechanical lysis. B. Trypsin method. C. Saponin method. Data are expressed as median and range. Labeled medians with identical letters are not significant. Different letters mean significant differences (*p* < 0.05) and were calculated using a Kruskal–Wallis test corrected by Bonferroni post hoc test. (**B**) A. Mechanical lysis. B. Trypsin method. Data are expressed as median and range. Labeled medians with identical letters are not significant. Different letters mean significant differences (*p* < 0.05) and were calculated using a Kruskal–Wallis test corrected by Bonferroni post hoc test.

**Table 2:** Identification of bacterial DNA based on shotgun using two extraction methods.

Variables	Control				Patients with Breast Cancer			
	Feces		Breast		Feces		Breast	
	A (n = 5)	B (n = 5)	A (n = 5)	B (n = 5)	A (n = 5)	B (n = 5)	A (n = 5)	B (n = 5)
*Actinomycetota*	0 (0–21.8)	1.9 (0–16.0) *	0 (0–0)	1.2 (1.0–1.0)	0.3 (0–26.3)	6.7 (2.0–14.0) *	0 (0–0)	0.7 (1.0–1.0)
*Bacteroidota*	0 (0–5.5)	4.2 (0–21.0) *	0 (0–0)	10.4 (10.0–10.0)	5.5 (0–39.2)	7.7 (1.0–28.0)	0 (0–0)	13.4 (12–15)
*Bacillota*	88.3 (72.6–100)	83.9 (63–100)	0 (0–0)	94.2 (88.0–100.0)	77.5 (46.8–100)	83.2 (56–100)	0 (0–0)	83.9 (80–88)
*Pseudomonadota*	0 (0–0)	1.7 (0–3.0) *	0 (0–0)	0 (0–0)	0 (0–6.7)	2.7 (1.0–5.0) *	0 (0–0)	4.7 (5.0–5.0)

Data are expressed as median and range. Labeled medians with * are significantly different (*p* < 0.05) and were calculated using a Kruskal–Wallis test corrected by Bonferroni post hoc test. **A.** Mechanical lysis. **B.** Trypsin method.

## Data Availability

Data are available on request from the authors. The data that support the findings of this study are available from the corresponding author upon reasonable request.
